# Supplementary figures and images for: Shoulder mobility and strength impairments in patients with rotator cuff related shoulder pain: a systematic review and meta analysis
Source: PeerJ. 2024 Jun 26;12:e17604. doi: 10.7717/peerj.17604 (PMC11214432; doi:10.7717/peerj.17604)

Standard Error

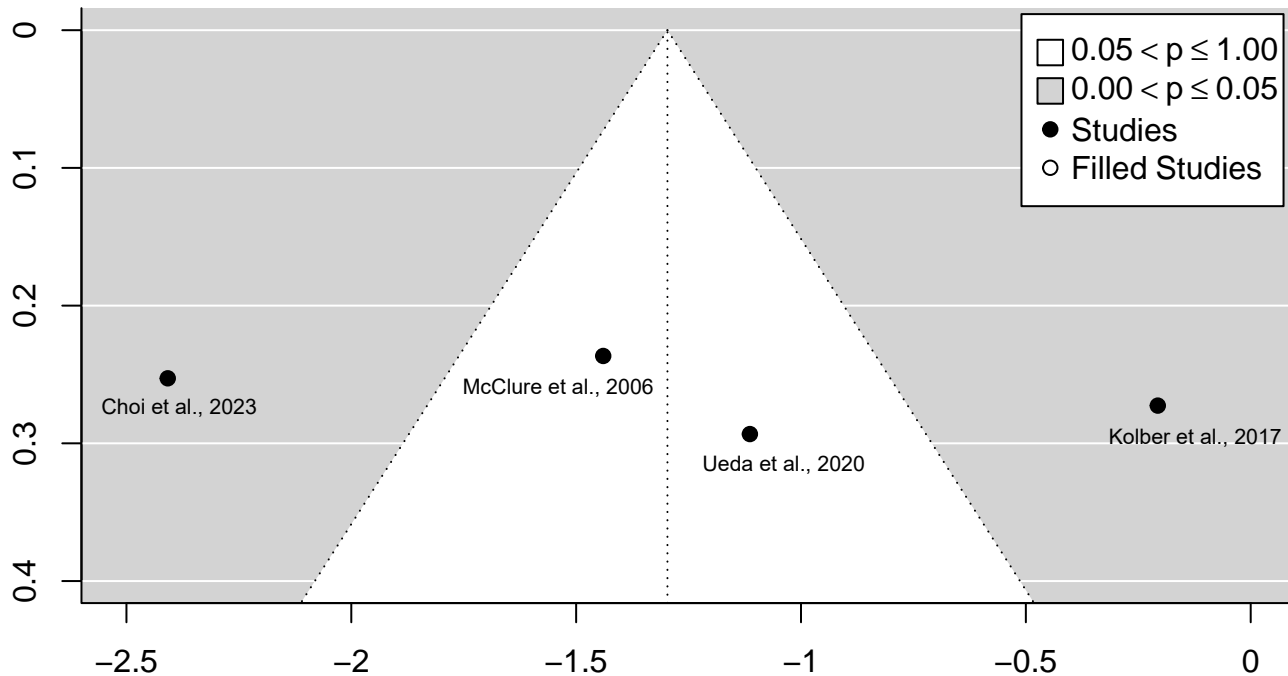

Standardized Mean Difference

Supplement: Supplemental Information 1 [file peerj-12-17604-s001.pdf]

Standard Error

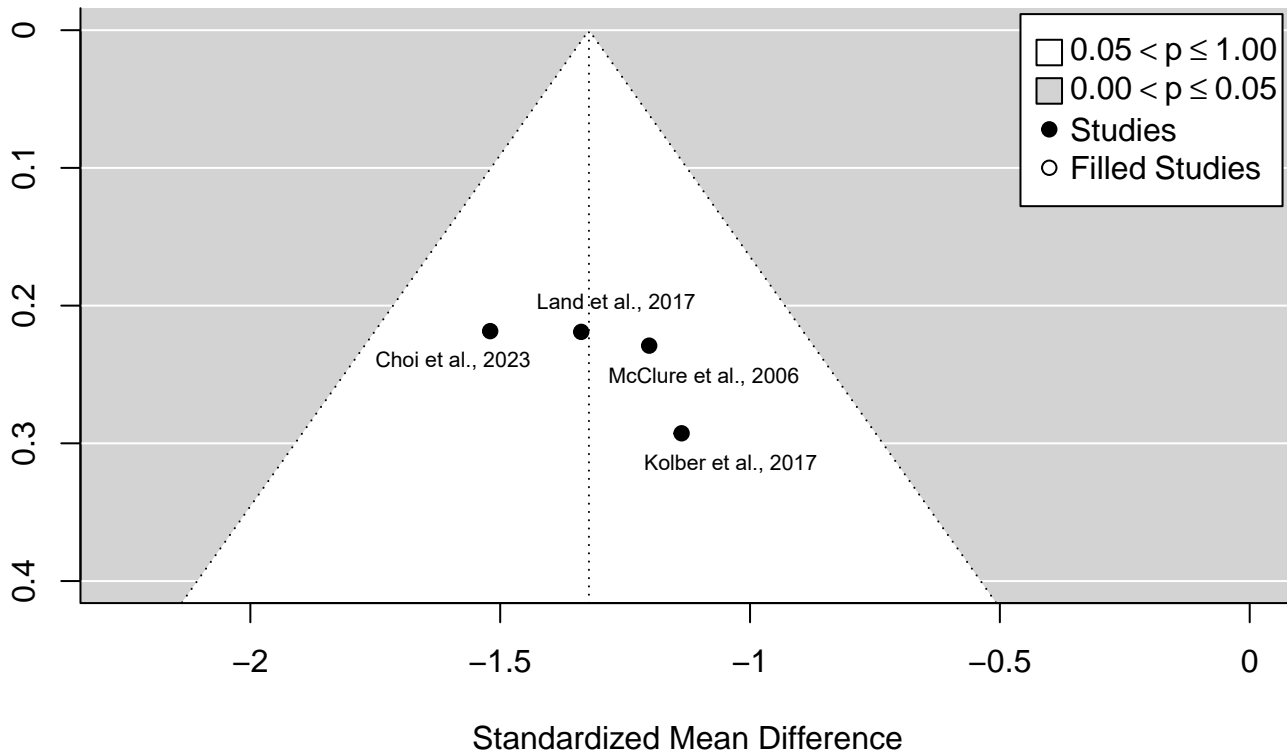

Supplement: Supplemental Information 2 [file peerj-12-17604-s002.pdf]

Standard Error

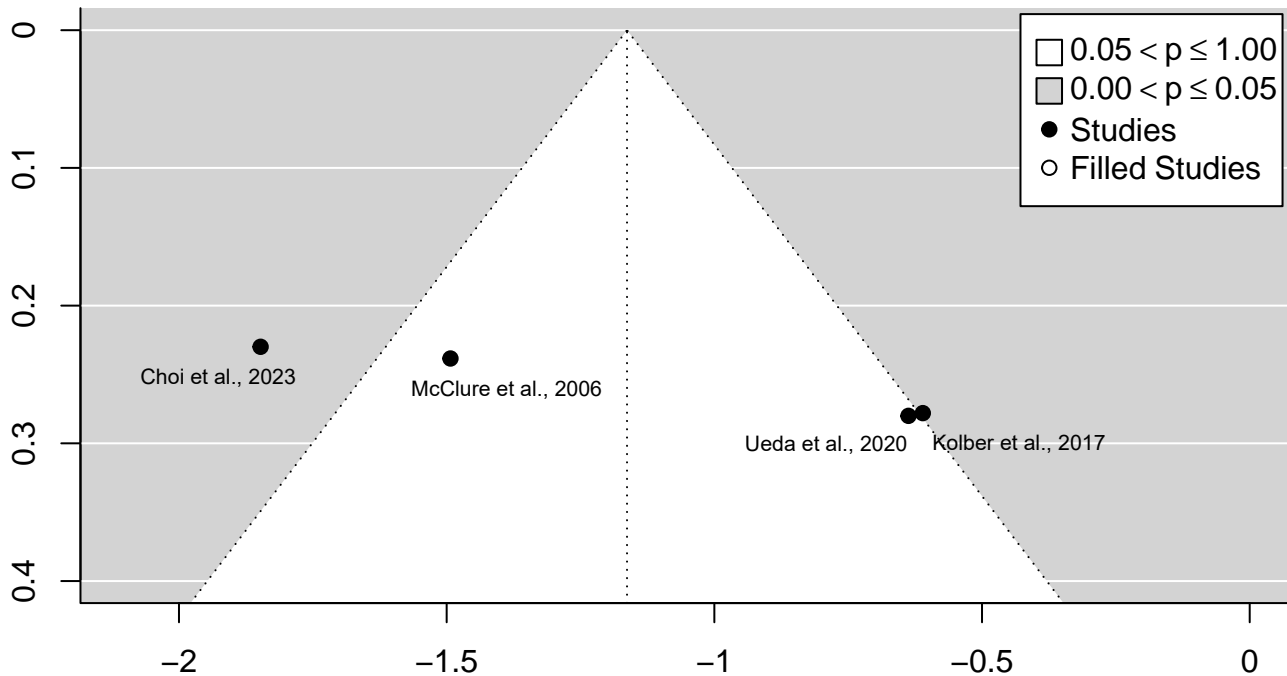

Standardized Mean Difference

Supplement: Supplemental Information 3 [file peerj-12-17604-s003.pdf]

Standard Error

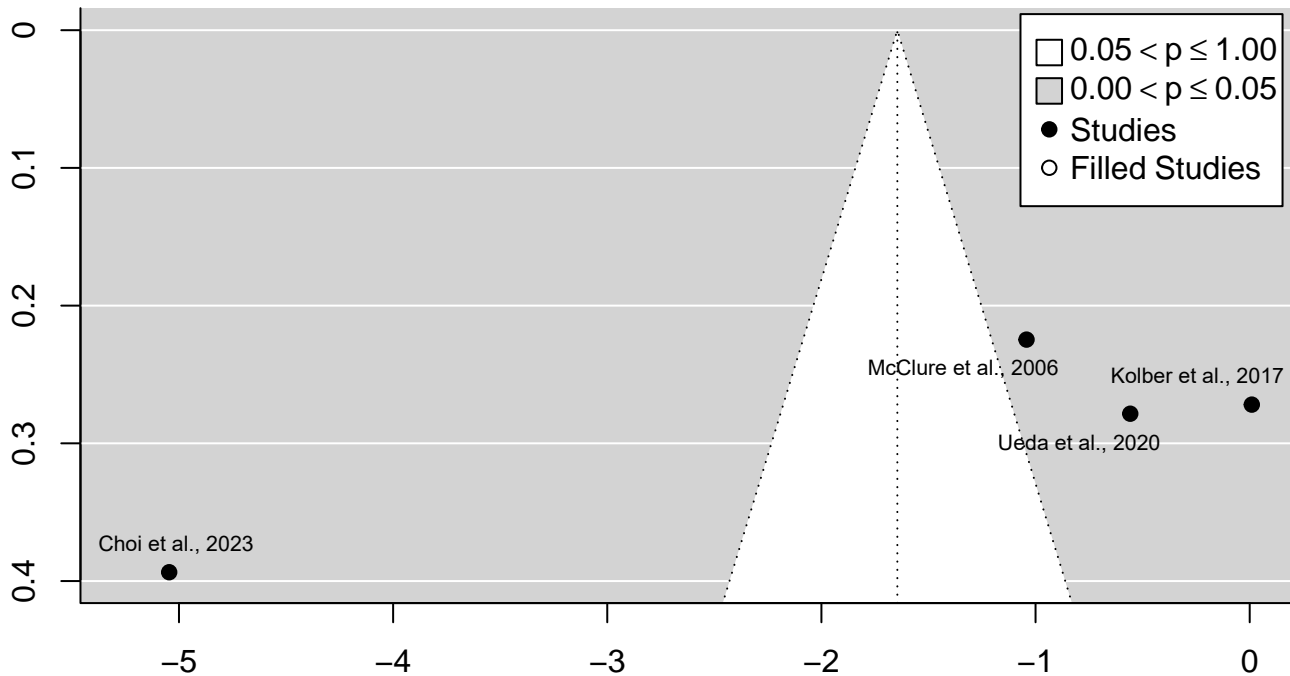

Standardized Mean Difference

Supplement: Supplemental Information 4 [file peerj-12-17604-s004.pdf]

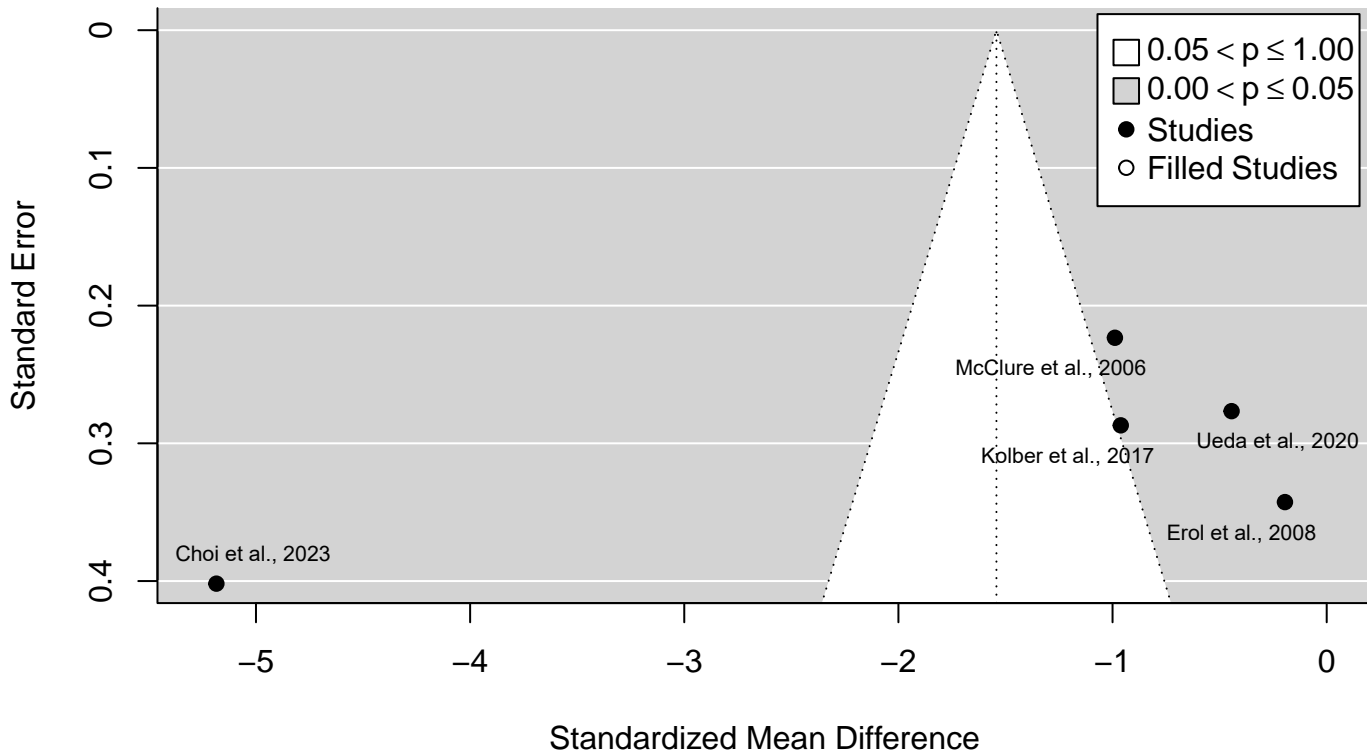

Supplement: Supplemental Information 5 [file peerj-12-17604-s005.pdf]

Standard Error

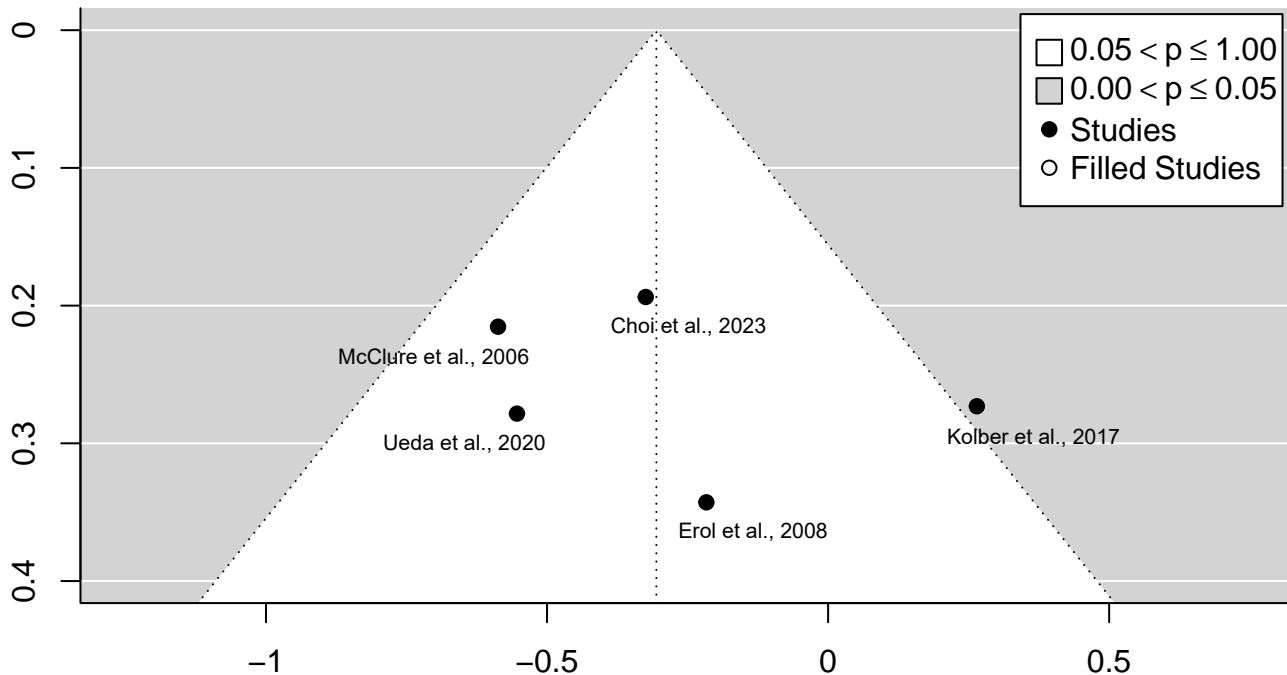

Standardized Mean Difference

Supplement: Supplemental Information 6 [file peerj-12-17604-s006.pdf]

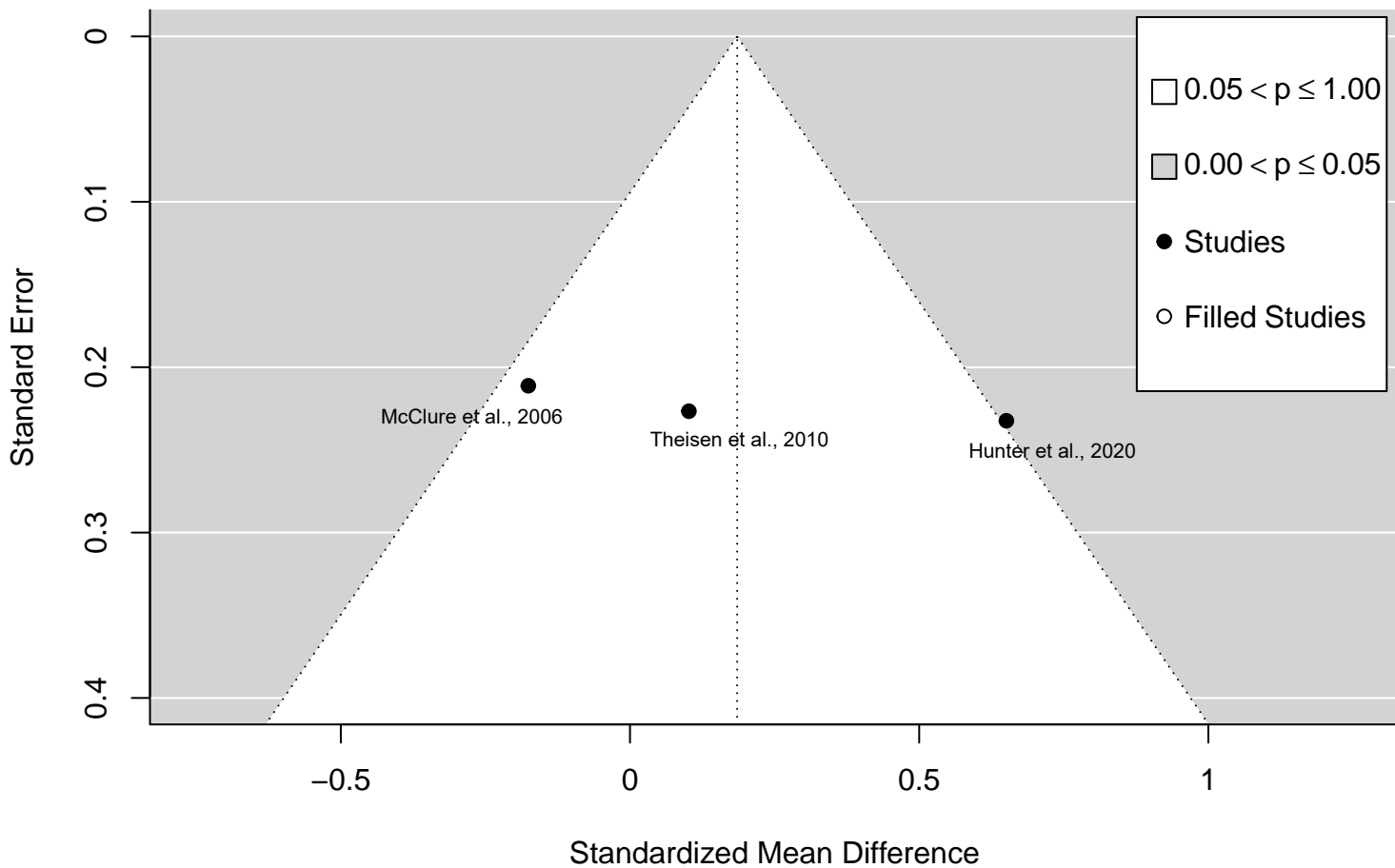

Supplement: Supplemental Information 7 [file peerj-12-17604-s007.pdf]
