# Supplementary material for: Shoulder mobility and strength impairments in patients with rotator cuff related shoulder pain: a systematic review and meta analysis
Source: PeerJ. 2024 Jun 26;12:e17604. doi: 10.7717/peerj.17604 (PMC11214432; doi:10.7717/peerj.17604)
Supplement: Supplemental Information 9 [file peerj-12-17604-s009.docx]

1. The rationale for conducting the systematic review/meta-analysis

Rotator cuff pathology is considered one of the most common causes of shoulder pain and disability, affecting millions of people worldwide. The term historically used to describe this condition was subacromial impingement syndrome, however, recent publications have challenged this diagnosis. A series of clinical terms have been proposed to actively move away from the term subacromial impingement syndrome. Rotator cuff-related shoulder pain (RCRSP) is an umbrella term that encompasses a spectrum of shoulder conditions including subacromial pain syndrome, subacromial impingement syndrome, rotator cuff tendinopathy, bursitis, and symptomatic partial and full thickness rotator cuff tears. Physical examination based on special orthopedic tests to identify the source of shoulder pain has been shown invalid. Recent studies concluded that the diagnosis of RCRSP should be based on the presence of pain during shoulder ABD and ER and the role of the thoracic spine as a source or contributing factor has been equally proposed. Hence, we considered that a systematic review and meta-analysis of shoulder range of motion, strength, and thoracic kyphosis in patients with RCRSP could provide a clinician useful information for a more evidence-based physical examination.

1. The contribution that it makes to knowledge in light of previously published related reports, including other meta-analyses and systematic reviews.

Traditionally, orthopedic shoulder tests and later shoulder strength have been proposed to assess patients with RCRSP. In this meta-analysis, we highlight the importance of also including the shoulder range of motion as statistical differences have been found in shoulder flexion and external and internal rotation.

To the best knowledge of the authors of the present study, no previous systematic review or meta-analysis has analyzed the differences in shoulder range of motion, shoulder strength, and thoracic kyphosis between RCRSP patients and an asymptomatic group.
